# Phase Ib Trial of Finotonlimab Combined With SCT200 in Patients With Recurrent or Metastatic Head and Neck Squamous Cell Carcinoma

**DOI:** 10.1002/cam4.72272

**Published:** 2026-09-07

**Authors:** Jinguan Lin, Song Qu, Kunyu Yang, Tao Zhang, Yuansong Bai, Jian Wu, Yanjing Huang, Meiyu Fang, Xianling Liu, Xiaoming Huang, Nianyong Chen, Zhendong Li, Weidong Li, Songnan Zhang, Shurong Zhang, Guangyuan Hu, Yan Sun, Xiaohong Chen, Yanyan Liu, Shanghua Jing, Liangfang Shen, Zhiwei Chang, Liangzhi Xie, Wenlin Gai, Yan Wang, Xinyue Chen, Dongfang Liu, Ye Guo

**Affiliations:** ^1^ The Affiliated Cancer Hospital of Xiangya School of Medicine Central South University/Hunan Cancer Hospital Changsha China; ^2^ Guangxi Medical University Cancer Hospital Nanning China; ^3^ Union Hospital, Tongji Medical College Huazhong University of Science and Technology Wuhan China; ^4^ The First Affiliated Hospital of Chongqing Medical University Chongqing China; ^5^ The Third Bethune Hospital of Jilin University Changchun China; ^6^ Chongqing University Cancer Hospital Chongqing China; ^7^ Hainan General Hospital Haikou China; ^8^ Zhejiang Cancer Hospital Hangzhou China; ^9^ The Second Xiangya Hospital of Central South University Changsha China; ^10^ Sun Yat‐sen Memorial Hospital Sun Yat‐sen University Guangzhou China; ^11^ West China Hospital Sichuan University Chengdu China; ^12^ Liaoning Cancer Hospital & Institute Shenyang China; ^13^ Affiliated Cancer Hospital and Institute of Guangzhou Medical University Guangzhou China; ^14^ Yanbian University Hospital Yanji China; ^15^ Beijing Tongren Hospital Affiliated to Capital Medical University Beijing China; ^16^ Tongji Hospital, Tongji Medical College Huazhong University of Science and Technology Wuhan China; ^17^ Peking University Cancer Hospital Beijing China; ^18^ Henan Cancer Hospital Zhengzhou China; ^19^ The Fourth Hospital of Hebei Medical University Shijiazhuang China; ^20^ Xiangya Hospital of Central South University Changsha China; ^21^ The First Affiliated Hospital of Zhengzhou University Zhengzhou China; ^22^ Beijing Engineering Research Center of Protein and Antibody Sinocelltech Ltd. Beijing China; ^23^ Shanghai East Hospital, School of Medicine Tongji University Shanghai China

**Keywords:** combination therapy, epidermal growth factor receptor, immunotherapy, recurrent or metastatic head and neck squamous cell carcinoma

## Abstract

**Background:**

Effective treatment options remain limited for patients with recurrent or metastatic head and neck squamous cell carcinoma (R/M HNSCC), including patients who have progressed after platinum‐based chemotherapy and systemic‐therapy‐naïve patients. This Phase Ib trial evaluates the efficacy and safety of finotonlimab, a PD‐1 antibody, combined with SCT200, an EGFR antibody.

**Methods:**

This prospective, multicenter, open‐label study enrolled patients into two cohorts: Cohort A (previously treated with platinum‐based chemotherapy and immune checkpoint inhibitors) and Cohort B (systemic therapy‐naïve). Patients received finotonlimab (200 mg every 3 weeks) and SCT200 (6 mg/kg weekly for 12 weeks, then 8 mg/kg every 2 weeks). The primary endpoint was objective response rate (ORR).

**Results:**

Among 41 patients, Cohort A (*n* = 11) showed an ORR of 27.3%, median progression‐free survival (PFS) of 5.8 months, and median overall survival (OS) of 10.6 months. Cohort B (*n* = 30) had an ORR of 56.7%, median PFS of 9.6 months, and an estimated median OS of 13.6 months. Common treatment‐related adverse events included hypomagnesemia (63.4%), rash (41.5%), and acneiform dermatitis (36.6%), which were manageable.

**Conclusions:**

The combination of finotonlimab and SCT200 demonstrates promising efficacy, particularly in systemic therapy‐naïve patients, warranting further investigation.

**Trial Registration:**

ClinicalTrials.gov identifier: NCT05552807; Chinadrugtrials.org.cn identifier: CTR20220917

## 1 Introduction

Head and neck squamous cell carcinoma (HNSCC) is a prevalent and aggressive malignancy, often linked to risk factors such as tobacco use, alcohol consumption, and human papillomavirus infection [1, 2]. Patients with recurrent or metastatic (R/M) HNSCC that cannot be treated with curative‐intent therapies have a poor prognosis, despite advancements in therapeutic approaches.

In recent years, immune checkpoint inhibitors (ICIs) have significantly improved treatment outcomes for patients with R/M HNSCC and have been widely adopted. Findings from the KEYNOTE‐048 trial indicate that the programmed cell death protein 1 (PD‐1) inhibitor pembrolizumab, both alone and in combination with chemotherapy, demonstrates survival benefits compared to cetuximab combined with chemotherapy in previously untreated R/M HNSCC patients. Pembrolizumab combined with chemotherapy significantly extends overall survival (OS) across all levels of programmed death ligand 1 (PD‐L1) combined positive score (CPS), with a median OS of 13.0 months, while pembrolizumab monotherapy extends OS specifically in patients with a PD‐L1 CPS ≥ 1, with a median OS of 12.3 months (CPS ≥ 1) [3, 4]. However, for previously untreated patients with low PD‐L1 CPS and for those who progress after platinum‐based chemotherapy and PD‐1 blockade, effective and better‐tolerated options remain limited. After the failure of PD‐1 inhibitor therapy, commonly used treatment options include monotherapies such as paclitaxel, docetaxel, and methotrexate, as well as cetuximab. However, their effectiveness is very limited, with a median OS of approximately 4.1–6.5 months [5, 6, 7, 8, 9].

To determine more effective and less toxic treatments, researchers have explored the molecular mechanisms underlying the synergistic effects of immunotherapy and targeted therapy and have attempted to combine PD‐1 antibodies with epidermal growth factor receptor (EGFR) antibodies. EGFR antibodies can trigger antibody‐dependent cellular cytotoxicity (ADCC), leading to interactions among immune cells such as natural killer cells and dendritic cells, while PD‐1 antibodies enhance the activity of cytotoxic lymphocytes, aiding in tumor regression and immune rejection [10, 11, 12, 13, 14]. The combination of adaptive and innate immunity with ADCC may result in enhanced antitumor effects. Two US Phase II trials have combined cetuximab with either pembrolizumab or nivolumab. The combination of cetuximab with pembrolizumab resulted in a median OS of 18.4 months with a response rate of 45%, while the combination of cetuximab with nivolumab resulted in a median OS of 20.2 months with a response rate of 37%. These findings are very encouraging [15, 16].

Finotonlimab (SCT‐I10A) and SCT200 are developed by Sinocelltech Ltd. Finotonlimab is a humanized IgG4 monoclonal antibody engineered to target PD‐1. In a Phase III study, finotonlimab, when combined with chemotherapy, significantly prolonged OS in the first‐line treatment of R/M HNSCC, with a median OS of 14.1 months [17]. SCT200 is a fully humanized IgG1 anti‐EGFR monoclonal antibody with a distinct antigen‐binding epitope, physicochemical properties, and biological activity compared to currently marketed anti‐EGFR monoclonal antibodies. It has demonstrated antitumor activity and a manageable safety profile in metastatic colorectal cancer [18, 19]. We hypothesized that the combination of finotonlimab and SCT200 would provide synergistic cancer control, and we assessed its efficacy and safety in R/M HNSCC patients both with and without prior exposure to systemic therapy.

## 2 Materials and Methods

### 2.1 Study Design and Participants

We conducted a prospective, multicenter, open‐label, multi‐arm Phase Ib study in 24 institutions in China to evaluate the efficacy and safety of finotonlimab in combination with SCT200 in R/M HNSCC. Cohort A comprised patients who had received prior systemic platinum‐based chemotherapy together with an immune checkpoint inhibitor (PD‐1/PD‐L1 antibody) and had progressed or were intolerant; these systemic therapies had been given either as curative‐intent multimodal treatment for locally advanced disease or as palliative therapy for R/M disease. Cohort B comprised patients who had received no prior systemic therapy for R/M disease; any prior treatment had been curative‐intent multimodal therapy (surgery, radiotherapy, and/or concurrent chemoradiation) for locally advanced disease, with recurrence documented more than 6 months after completing that treatment, and these patients were therefore systemic‐therapy‐naïve and platinum‐sensitive. Neither cohort had previously received EGFR monoclonal antibodies.

This trial comprised four arms under a single protocol (ClinicalTrials.gov NCT05552807): Cohort 1 (prior platinum‐based therapy, immune checkpoint inhibitor–naïve), Cohort 2 (prior platinum plus immune checkpoint inhibitor, randomly assigned 1:1 to Cohort 2‐1 and Cohort 2‐2), and Cohort 3 (systemic‐therapy‐naïve, recurrence > 6 months after curative‐intent therapy). Cohorts 1, 2‐1, and 3 received finotonlimab plus SCT200, whereas Cohort 2‐2 received SCT200 plus paclitaxel or docetaxel. The present report describes the two finotonlimab‐plus‐SCT200 arms enrolled during the study period: Cohort A (protocol Cohort 2‐1) and Cohort B (protocol Cohort 3). The SCT200‐plus‐paclitaxel/docetaxel arm (protocol Cohort 2‐2) has been reported separately [20]; Cohort 1 is not reported here. The two publications describe non‐overlapping patients.

Additional key inclusion criteria were: age 18 years or older; an Eastern Cooperative Oncology Group (ECOG) performance status of 0 or 1; histologically confirmed squamous cell carcinoma of the head and neck with distant metastasis or recurrence not amenable to curative locoregional treatment; and measurable disease as defined by the Response Evaluation Criteria in Solid Tumors (RECIST) version 1.1 (assessed by investigator review). Patients were also required to have adequate hepatic, renal, and bone marrow function and to provide written informed consent.

### 2.2 Procedures

Participants received a fixed dose of 200 mg finotonlimab intravenously on the first day of each 3‐week cycle and SCT200 at 6 mg/kg intravenously once weekly for the first 12 weeks, followed by 8 mg/kg intravenously once every 2 weeks, until disease progression, unacceptable toxicity, or a decision by the patient or investigator to terminate treatment. Physical examinations, adverse‐event assessments, and routine bloodwork were performed at study entry and before each cycle. Tumor response was assessed by computed tomography or magnetic resonance imaging within 28 days before the initial treatment (baseline) and every 6 weeks (±7 days) during treatment, regardless of treatment delays or interruptions. An end‐of‐treatment visit was conducted within 7 days after the last dose. Participants who discontinued treatment entered survival follow‐up and were monitored every 12 weeks (±14 days).

### 2.3 Outcomes

The primary endpoint was the objective response rate (ORR), defined as the proportion of participants achieving a complete response (CR) or partial response (PR) in accordance with RECIST version 1.1. Secondary endpoints included progression‐free survival (PFS), defined as the time from initial study‐drug administration to disease progression or death from any cause; OS, defined as the time from initial study‐drug administration to death from any cause; disease control rate (DCR), defined as the proportion with CR, PR, or stable disease (SD); duration of response (DOR), defined as the time from documentation of tumor response to disease progression or death; and safety and tolerability. All tumor response assessments were performed by the investigators without blinded independent central review. Adverse events (AEs) were graded according to the Common Terminology Criteria for Adverse Events (CTCAE) version 5.0.

### 2.4 Statistical Analysis

Given the exploratory nature of this Phase Ib study, formal sample‐size calculation was not performed, and no target ORR was pre‐specified in the study protocol. Before patient enrollment, based on previously published findings of PD‐1‐inhibitor combined with EGFR‐antibody for R/M HNSCC, we assumed that this combination could yield clinically meaningful antitumor activity in our study populations. The planned accrual was 30 patients for each cohort, following conventional practice for early‐phase oncology exploratory trials. For efficacy assessment, tumor response endpoints were analyzed separately for each cohort, and no cross‐cohort pooled analyses of response data were performed. The primary analysis was conducted after the last patient had been followed for more than 3 months post‐enrollment. Analyses for primary and secondary endpoints were performed in patients who had received at least one dose of study drug. Demographic and baseline characteristics were described using appropriate statistical methods. ORR and DCR were summarized descriptively. The Kaplan–Meier method was used to estimate median OS, PFS, and DOR with 95% confidence intervals (CIs). Safety and tolerability endpoints for Cohort A and Cohort B were analyzed together and described using descriptive statistics. Analyses were performed using SAS version 9.4.

## 3 Results

### 3.1 Patient Characteristics

**FIGURE 1:**
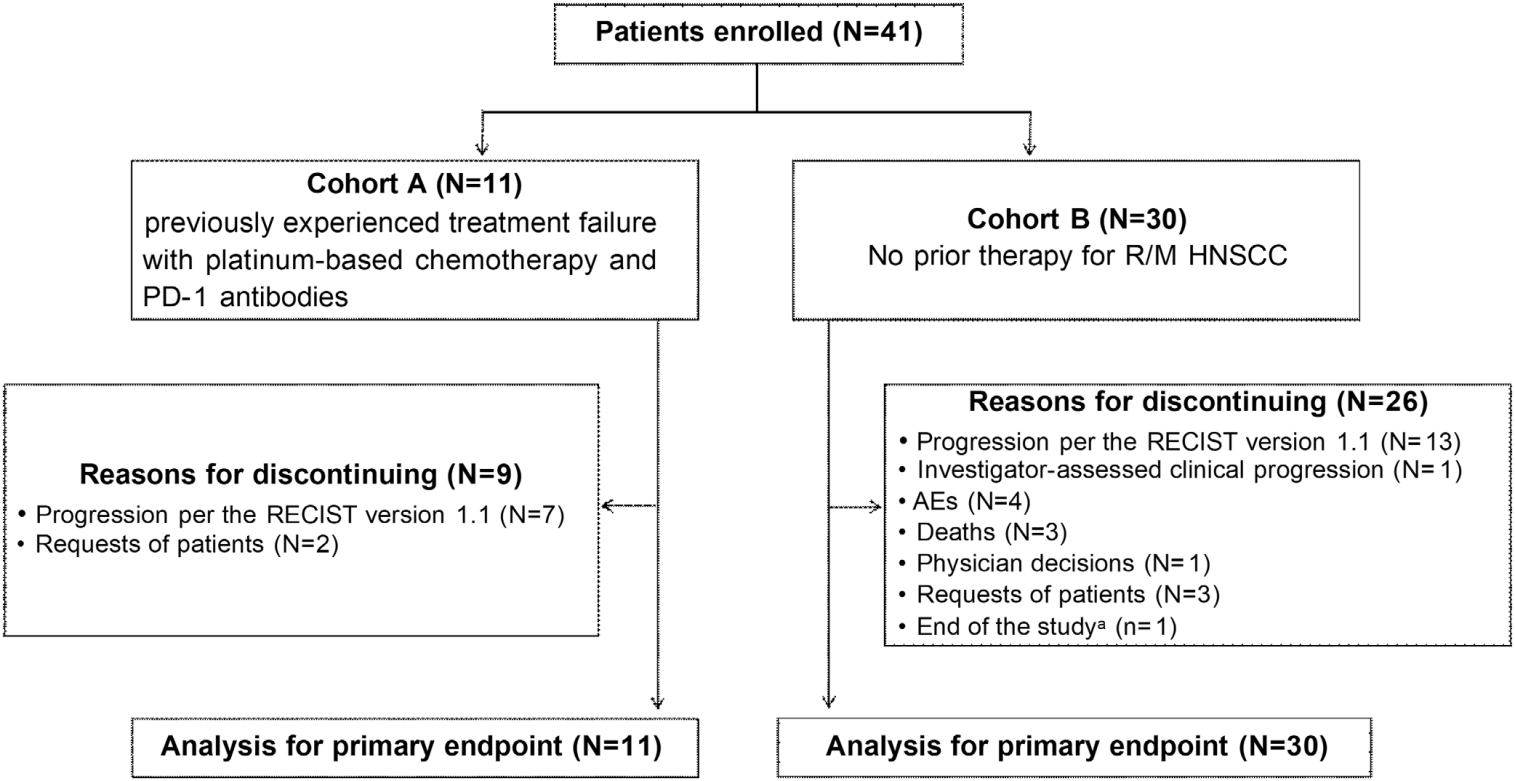
Trial profile. Enrolled patients (*n* = 41) by cohort and reasons for discontinuation. ᵃOne patient in Cohort B discontinued due to the end of the study for a reason other than disease progression or intolerable toxicity.

Between June 2022 and December 2023, 41 patients met the eligibility criteria and were enrolled (Figure 1). Screening and screen‐failure data were recorded at the trial level across all four cohorts, without an intended‐cohort field at screening; screen failures therefore cannot be partitioned by cohort. Because this report presents two of the four cohorts, and the screened population also gave rise to patients enrolled in the two cohorts not reported here, a screening‐to‐enrolment flow terminating at the 41 Cohort A/B patients cannot be constructed without misrepresenting the denominator; Figure 1 therefore begins at enrolment, and this constraint is noted among the study limitations. All 41 patients received at least one dose of study drug. Eleven patients were assigned to Cohort A; because only pembrolizumab and nivolumab had been approved by the China National Medical Products Administration at the time the trial commenced, and both were prohibitively expensive, the number of eligible patients was limited, and enrollment into Cohort A was terminated early (11 of the planned 30). The remaining 30 participants were enrolled into Cohort B per the protocol.

Detailed baseline characteristics are provided in Table 1. The proportion of patients with an ECOG performance status of 0 was low in both cohorts (9.1% and 16.7%). All patients in Cohort A had previously received both platinum‐based chemotherapy and PD‐1 antibody therapy, with more than half having undergone at least two lines of treatment before study entry. The types of prior PD‐1/PD‐L1 agents for Cohort A are summarized in Table S1, from which the duration of prior therapy can be derived. Best response to prior immunotherapy and the treatment‐free interval before study entry could not be reliably obtained, as these were held only in external medical records; this is acknowledged as a limitation. All patients in Cohort B had received no prior systemic therapy in the recurrent or metastatic setting. The data cut‐off was July 29, 2024. The median follow‐up from initiation of study treatment to data cutoff or death was 15.6 months (95% CI, 15.2 to not reached) for Cohort A and 14.0 months (95% CI, 12.2–14.8) for Cohort B. At analysis, 2 patients (18.2%) in Cohort A and 4 patients (13.3%) in Cohort B were continuing study treatment.

**TABLE 1:** Baseline demographic and disease characteristics.

	Cohort A (*N* = 11), *n* (%)	Cohort B (*N* = 30), *n* (%)
Age (years)		
Median (min, max)	55 (39, 60)	58 (33, 77)
Sex		
Male	8 (72.7)	29 (96.7)
Female	3 (27.3)	1 (3.3)
ECOG PS score		
0	1 (9.1)	5 (16.7)
1	10 (90.9)	25 (83.3)
CPS		
≥ 20	3 (27.3)	5 (16.7)
≥ 1	7 (63.6)	18 (60.0)
< 1	3 (27.3)	8 (26.7)
Not evaluated	1 (9.1)	4 (13.3)
Primary site		
Oral cavity	5 (45.5)	17 (56.7)
Oropharynx	3 (27.3)	3 (10.0)
Hypopharynx	2 (18.2)	4 (13.3)
Larynx	0	6 (20.0)
Others	1 (9.1)	0
Disease stage		
Metastasis	7 (63.6)	14 (46.7)
Relapse only	4 (36.4)	16 (53.3)
Previous treatment		
Surgery	7 (63.6)	25 (83.3)
Radiotherapy	10 (90.9)	13 (43.3)
Line of prior therapy for R/M HNSCC		
Median (min, max)	2 (1, 3)	0
1	5 (45.5)	0
2	4 (36.4)	0
≥ 3	2 (18.2)	0

*Note:* CPS categories are mutually exclusive (≥ 20 is a subset of ≥ 1); “Not evaluated” denotes patients without an available CPS result.

Abbreviations: CPS, combined positive score; ECOG PS, Eastern Cooperative Oncology Group performance status; *N*/*n*, number; R/M HNSCC, recurrent and metastatic head and neck squamous cell carcinoma.

### 3.2 Efficacy for Cohort A

**FIGURE 2:**
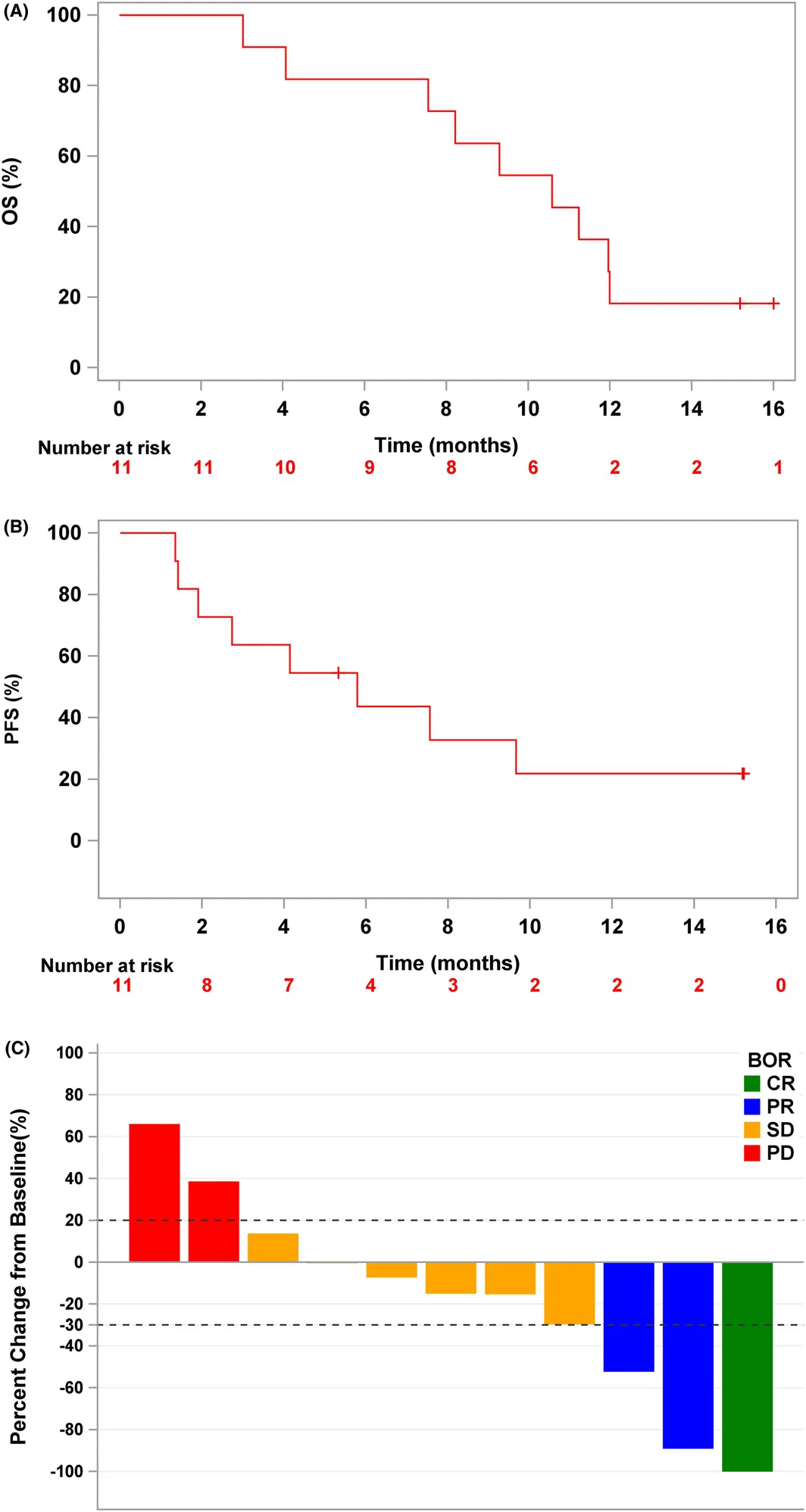
Survival outcomes and best response for Cohort A. (A) Kaplan–Meier curve of OS; (B) Kaplan–Meier curve of PFS (vertical ticks denote censored data); (C) waterfall plot of best percentage change from baseline in target lesions (dashed lines: 30% reduction and 20% increase).

The ORR was 27.3% (95% CI, 6.02–60.97), with 1 CR and 2 PRs in this heavily pretreated cohort, all of whom had previously failed platinum‐based chemotherapy and PD‐1 antibodies. Six patients had SD, giving a DCR of 81.8% (95% CI, 48.22–97.72). Among patients achieving a complete or partial response, the median DOR was not reached (95% CI, 6.9 months to not reached). The median PFS was 5.8 months (95% CI, 1.4 to not reached), and the median OS was 10.6 months (95% CI, 4.1–12.0) (Figure 2 and Table S2).

### 3.3 Efficacy for Cohort B

The efficacy assessment included all 30 enrolled patients; 3 patients were not evaluable (2 without post‐baseline imaging and 1 who received additional antitumor therapy of unknown start date; Table S2). The ORR was 56.7% (95% CI, 37.43–74.54) and the DCR was 86.7% (95% CI, 69.28–96.24). Among patients achieving a complete or partial response, the median DOR was 8.4 months (95% CI, 4.1 months to not reached), and the median PFS was 9.6 months (95% CI, 4.0–12.4). With 15 deaths (50%) at data cutoff, the estimated median OS was 13.6 months, which remained immature (upper bound not reached).

**FIGURE 3:**
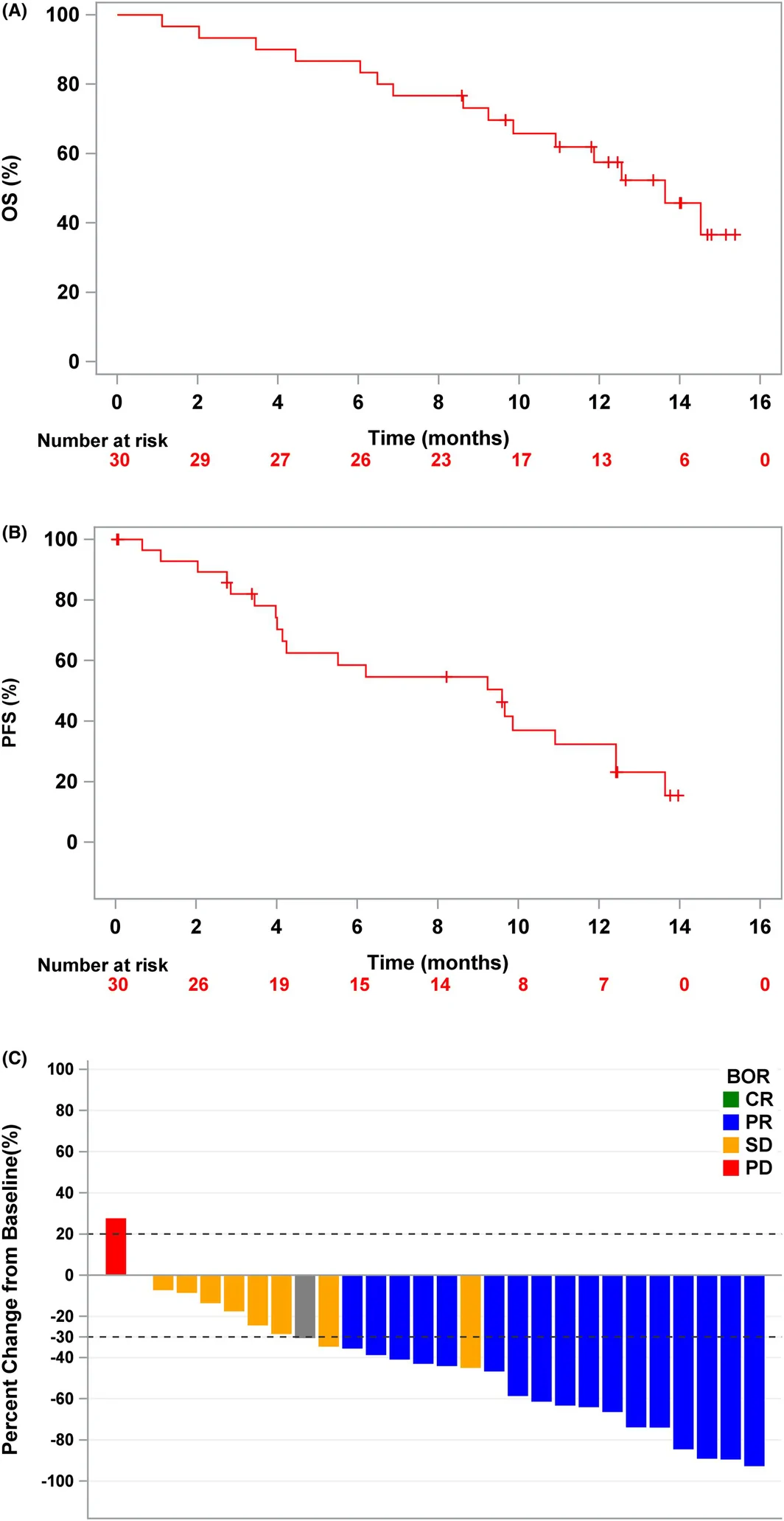
Survival outcomes and best response for Cohort B. (A) Kaplan–Meier curve of OS; (B) Kaplan–Meier curve of PFS (vertical ticks denote censored data); (C) waterfall plot of best percentage change from baseline in target lesions. One patient received additional antitumor therapy outside the study, with an unknown start date potentially preceding the response evaluation and was classified as not evaluable.

Baseline CPS is reported for both cohorts in Table 1. Because of the small size of Cohort A, CPS‐stratified efficacy was explored only in Cohort B, and CPS was not evaluable in all patients (CPS < 1, *n* = 8; CPS ≥ 1, *n* = 18). The ORR, median PFS, and median OS for patients with CPS < 1 versus CPS ≥ 1 were 50.0% (95% CI, 15.70–84.30) versus 72.2% (95% CI, 46.52–90.31); 12.4 months (95% CI, 3.4 to not reached) versus 9.6 months (95% CI, 4.0–12.4); and not reached (95% CI, 3.4 to not reached) versus 14.5 months (95% CI, 11.9 to not reached), respectively. Although patients with CPS ≥ 1 had a numerically higher ORR than those with CPS < 1, both median PFS and median OS were numerically shorter in the CPS ≥ 1 subgroup. Given the small subgroups, incomplete CPS evaluation, and immature OS, no formal statistical comparison was performed. With subgroups this small and survival still immature, the CPS pattern is unstable, and we draw no inference from it. At the cutoff, 15 deaths (50%) had occurred, 4 patients (13.3%) were continuing study treatment, 1 patient (3.3%) was lost to follow‐up, and 10 patients (33.3%) were alive in survival follow‐up (Figure 3 and Table S2).

### 3.4 Safety

The safety population comprised all 41 patients. Every patient experienced treatment‐emergent adverse events, and 40 patients (97.6%) experienced treatment‐related adverse events (TRAEs). The most common TRAEs of any grade were hypomagnesemia (63.4%, 26/41), rash (41.5%, 17/41), and acneiform dermatitis (36.6%, 15/41). Grade 3 or 4 TRAEs occurred in 25 of 41 patients (61.0%); the most frequent (in ≥ 2 patients) were hypomagnesemia (39.0%, 16/41), rash (9.8%, 4/41), hypocalcemia (7.3%, 3/41), hypokalemia (7.3%, 3/41), and acneiform dermatitis (4.9%, 2/41) (Table 2). Grade ≥ 3 TRAEs attributed to each study drug are shown in Tables S3 (finotonlimab) and S4 (SCT200); because an event could be judged related to more than one drug, these tables are not mutually exclusive and their totals do not sum to the overall number of grade ≥ 3 TRAEs.

**TABLE 2:** Treatment‐related adverse events (TRAEs) occurring in ≥ 10% of patients (finotonlimab plus SCT200).

Adverse event	Any grade, *n* (%)	Grade 3 or 4, *n* (%)
Any TRAE	40 (97.6)	25 (61.0)
Hypomagnesemia	26 (63.4)	16 (39.0)
Rash	17 (41.5)	4 (9.8)
Acneiform dermatitis	15 (36.6)	2 (4.9)
Hypothyroidism	12 (29.3)	0
Aspartate aminotransferase increased	9 (22.0)	1 (2.4)
Paronychia	9 (22.0)	1 (2.4)
Anemia	9 (22.0)	1 (2.4)
Alanine aminotransferase increased	8 (19.5)	1 (2.4)
Infusion reaction	8 (19.5)	0
Hypoalbuminemia	6 (14.6)	0
Hypocalcemia	6 (14.6)	3 (7.3)
Oral mucositis	6 (14.6)	0
Maculopapular rash	5 (12.2)	1 (2.4)
Conjunctivitis	5 (12.2)	0
Proteinuria	5 (12.2)	0
Hematuria	5 (12.2)	0

*Note:* Percentages are based on the safety population (*N* = 41).

Abbreviations: *N*, number; TRAE, treatment‐related adverse event.

Two patients discontinued study treatment due to TRAEs: one discontinued both drugs due to Grade 4 diabetic ketoacidosis attributed to finotonlimab, and another discontinued SCT200 (continuing finotonlimab) due to Grade 3 paronychia related to SCT200. Infusion reactions were reported in eight patients (19.5%), all Grade 1 or 2. One occurred in Cohort A and was assessed as definitely related to SCT‐I10A and probably unrelated to SCT200; the remaining seven occurred in Cohort B and were rated as possibly related to both study drugs, so a single causative agent could not be assigned. Seven patients had reactions during the first infusion, and one had Grade 1 reactions during the first and second infusions; all resolved without recurrence. The AEs were manageable, and no treatment‐related deaths were observed (Table 2 and Table S5).

Grade ≥ 3 TRAEs were managed according to standard clinical practice. Electrolyte abnormalities were corrected with oral or intravenous repletion under serum monitoring, and SCT200 was held when a Grade 3 event persisted. Skin toxicity was treated with topical steroids and antibiotics, with systemic steroids added at Grade 3 or 4. The immune‐mediated events were the most serious: both the case of diabetic ketoacidosis and the case of interstitial lung disease required high‐dose corticosteroids, discontinuation of finotonlimab, and multidisciplinary care. Liver‐enzyme elevation, hypertension, and anemia were managed supportively, and infusion reactions with a slower infusion rate and antihistamine premedication.

### 3.5 Post‐Progression Treatments

After progression on finotonlimab plus SCT200, 7 of 11 patients (63.6%) in Cohort A and 11 of 30 patients (36.7%) in Cohort B received subsequent anti‐tumor therapy. In Cohort A, post‐progression regimens mainly comprised chemotherapy (36.4%), EGFR monoclonal antibodies (27.3%), PD‐1/PD‐L1 inhibitors (27.3%), and tyrosine‐kinase inhibitors (18.2%); 2 patients (18.2%) entered other clinical trials. In Cohort B (systemic‐therapy‐naïve), subsequent therapies were dominated by chemotherapy (23.3%) and PD‐1/PD‐L1 agents (13.3%). Detailed distributions are summarized in Table S6.

## 4 Discussion

This study provides insights for the development of treatment strategies for R/M HNSCC, especially for patients previously exposed to PD‐1 antibodies and for systemic‐therapy‐naïve patients. The immune activity of EGFR antibodies, including but not limited to ADCC, provides a rationale for their combination with ICIs to synergistically mobilize adaptive and innate immune responses against tumor cells [21]. ICIs may also enhance the efficacy of EGFR antibodies, potentially improving clinical outcomes [22].

In patients previously treated with platinum‐based chemotherapy, chemotherapy or cetuximab monotherapy has been standard, with reported ORR of 6.2%–14.0%, median PFS of 2.1–3.5 months, and median OS of 4.1–6.5 months [5, 6, 7, 8, 9]. In patients who failed both platinum‐based chemotherapy and ICIs, no standardized approach has been established. Although the utility of PD‐1 rechallenge remains debated in the context of primary, adaptive, and acquired resistance to immune checkpoint inhibitors [23], the combination in Cohort A demonstrated an ORR of 27.3%, median PFS of 5.8 months, and median OS of 10.6 months. These findings are consistent with the nivolumab‐plus‐cetuximab combination in R/M HNSCC (ORR 22.2%, median PFS 3.4 months, median OS 9.7 months); notably, prior ICI exposure was not required in that study, and some patients were ICI‐naïve [24].

For first‐line treatment of R/M HNSCC, KEYNOTE‐048 reported an ORR of 16.9%, median PFS of 2.3 months, and median OS of 11.5 months in the total population with pembrolizumab monotherapy, and an ORR of 36.3%, median PFS of 4.9 months, and median OS of 13.0 months with pembrolizumab plus chemotherapy, across the total population with any PD‐L1 CPS [3, 4]. Because Cohort B was systemic‐therapy‐naïve with recurrence more than 6 months after curative‐intent therapy, it represents a platinum‐sensitive, first‐line population aligned with the eligibility of KEYNOTE‐048. In Cohort B, the ORR was 56.7%, median PFS 9.6 months, and estimated median OS 13.6 months, although the OS estimate remains immature (median follow‐up 14.0 months). These comparisons are descriptive and cross‐trial, and the single‐arm design, the small sample, and investigator‐based assessment all limit how much weight they can bear. Two US Phase II studies (pembrolizumab or nivolumab plus cetuximab) reported ORR of 37%–45%, median PFS of 6.2–6.5 months, and median OS of 18.4–20.2 months [15, 16]; differences from our results may reflect variation in baseline characteristics and small‐sample bias.

No unexpected safety signals were observed; TRAEs were consistent with the known profiles of each drug. The most common and significant Grade 3 or 4 TRAEs were hypomagnesemia and cutaneous reactions, most of which resolved with symptomatic treatment or drug suspension. The profile of common TRAEs was comparable to cetuximab plus pembrolizumab or nivolumab [15, 16], while treatment discontinuation due to TRAEs was lower in our study (2/41 vs. 5/33 in the cetuximab‐plus‐pembrolizumab study) [15].

This study has several limitations. Owing to the exploratory, open‐label, single‐arm design and small sample size, the results may be biased, and larger studies are needed. PD‐L1 was not tested in all subjects, and human papillomavirus status and detailed anatomical subsite were not collected, restricting conclusions related to CPS, HPV, and subsite. Tumor responses were assessed by local investigators without blinded independent central review (BICR); investigator‐based assessment in an open‐label, single‐arm study may overestimate the ORR and cannot reliably distinguish true progression from immune‐related pseudo‐progression, and future trials with BICR are warranted. For Cohort A, best response to and treatment‐free interval after prior ICI were incompletely available from external records. Finally, because screening data were captured at the trial level for all four cohorts and could not be attributed to individual cohorts, a screening‐to‐enrolment flow could not be presented for the two cohorts reported here.

## 5 Conclusions

Finotonlimab plus SCT200 achieved a notable ORR and prolonged PFS and OS in R/M HNSCC, particularly in patients without prior systemic therapy in the recurrent or metastatic setting, and was well tolerated. These results may inform treatment strategies for systemic‐therapy‐naïve patients and for those with resistance to both platinum‐based chemotherapy and PD‐1 inhibitors, and support further investigation in larger, controlled studies.

## Author Contributions

**Guangyuan Hu:** investigation, resources. **Jian Wu:** investigation, resources. **Yanjing Huang:** investigation, resources. **Song Qu:** investigation, resources. **Jinguan Lin:** investigation, resources. **Yan Sun:** investigation, resources. **Meiyu Fang:** investigation, resources. **Tao Zhang:** investigation, resources. **Xiaohong Chen:** investigation, resources. **Liangzhi Xie:** conceptualization, methodology, project administration. **Xianling Liu:** investigation, resources. **Ye Guo:** investigation, resources, conceptualization, methodology, writing – review and editing. **Liangfang Shen:** investigation, resources. **Xiaoming Huang:** investigation, resources. **Zhiwei Chang:** investigation, resources. **Nianyong Chen:** investigation, resources. **Yuansong Bai:** investigation, resources. **Shanghua Jing:** investigation, resources. **Kunyu Yang:** investigation, resources. **Yanyan Liu:** investigation, resources. **Wenlin Gai:** conceptualization, methodology, project administration. **Songnan Zhang:** investigation, resources. **Zhendong Li:** investigation, resources. **Weidong Li:** investigation, resources. **Yan Wang:** conceptualization, methodology, project administration. **Dongfang Liu:** formal analysis, software. **Shurong Zhang:** investigation, resources. **Xinyue Chen:** writing – original draft, writing – review and editing, supervision, project administration.

## Funding

Sponsored by Sinocelltech Ltd., Beijing, China, and funded by the National Science and Technology Major Projects of China (2019ZX09732‐001).

## Ethics Statement

The study was approved by the Medical Ethics Committee of Shanghai East Hospital, Tongji University (no. [2021] Lin‐Shen 078) and by the ethics committee of each participating site, and was conducted in accordance with the Declaration of Helsinki and Good Clinical Practice.

## Consent

Written informed consent was obtained from all participants.

## Conflicts of Interest

The authors declare no conflicts of interest.

## Conflicts of Interest

Liangzhi Xie, Wenlin Gai, Yan Wang, Xinyue Chen, and Dongfang Liu are employees of Sinocelltech Ltd., Beijing, China. Ye Guo reports honoraria or consulting fees from Merck Serono, Roche, MSD, BeOne, AstraZeneca, Bristol Myers Squibb, and Johnson & Johnson outside the submitted work. The other authors, who are investigators involved in this study, declare no competing interests.

## Supporting information


**Table S1:** Prior anti‐PD‐(L)1 therapy in Cohort A.
**Table S2:** Efficacy endpoints of finotonlimab plus SCT200.
**Table S3:** Grade ≥ 3 TRAEs attributed to finotonlimab (SCT‐I10A).
**Table S4:** Grade ≥ 3 TRAEs attributed to SCT200.
**Table S5:** TRAEs occurring in ≥ 5% of patients (finotonlimab plus SCT200).
**Table S6:** Post‐progression anti‐cancer treatments after finotonlimab plus SCT200.

## Data Availability

The datasets generated and analyzed during the current study are not publicly available because they contain proprietary sponsor information and individual patient data subject to privacy protection, but are available from the corresponding author on reasonable request and with permission from the sponsor (Sinocelltech Ltd.).
