# Supplementary material for: Phase Ib Trial of Finotonlimab Combined With SCT200 in Patients With Recurrent or Metastatic Head and Neck Squamous Cell Carcinoma
Source: Cancer Med. 2026 Sep 7;15(9):e72272. doi: 10.1002/cam4.72272 (PMC13550516; doi:10.1002/cam4.72272)
Supplement: Supplementary file 1 [file CAM4-15-e72272-s001.docx]

**Supplementary Material**

*Phase Ib Trial of Finotonlimab Combined with SCT200 in Patients with Recurrent or Metastatic Head and Neck Squamous Cell Carcinoma*

**Table S1. Prior anti-PD-(L)1 therapy in Cohort A**

| **Subject** | **Prior anti-PD-(L)1** | **Start** | **End** |
| --- | --- | --- | --- |
| 02023 | Toripalimab | 2022-12-15 | 2023-04-12 |
| 04006 | Sintilimab | 2022-07-01 | 2022-10-09 |
| 05005 | SHR-1701 (anti-PD-L1/TGF-βRII) | 2021-07-09 | 2022-02-08 |
| 07001 | Pembrolizumab | 2022-04-30 | 2022-05-21 |
| 10001 | Pembrolizumab | 2022-02-10 | 2022-06-30 |
| 10003 | Nivolumab | 2022-07-02 | 2022-11-03 |
| 11002 | Pembrolizumab | 2022-10-26 | NA |
| 12001 | Toripalimab | 2022-10-15 | 2022-11-29 |
| 24003 | Camrelizumab | 2022-07-03 | 2022-08-24 |
| 25004 | Toripalimab | 2021-11-24 | 2022-04-19 |
| 25005 | Pembrolizumab | 2022-01-13 | 2022-03-15 |

NA, not available. The duration of prior therapy can be derived from the start and end dates.

**Table S2. Efficacy endpoints of finotonlimab plus SCT200**

|  | **Cohort A (N = 11)** | **Cohort B (N = 30)** |
| --- | --- | --- |
| **Best overall response, n (%)** |  |  |
| CR | 1 (9.1) | 0 |
| PR | 2 (18.2) | 17 (56.7) |
| SD | 6 (54.5) | 9 (30.0) |
| PD | 2 (18.2) | 1 (3.3) |
| NE ᵃ | 0 | 3 (10.0) |
| **ORR (CR+PR), n (%)** | 3 (27.3) | 17 (56.7) |
| 95% CI | 6.0, 61.0 | 37.4, 74.5 |
| **DCR (CR+PR+SD), n (%)** | 9 (81.8) | 26 (86.7) |
| 95% CI | 48.2, 97.7 | 69.3, 96.2 |
| **DOR, median (95% CI), months** | NR (6.9, NR) | 8.4 (4.1, NR) |
| **PFS, median (95% CI), months** | 5.8 (1.4, NR) | 9.6 (4.0, 12.4) |
| **OS, median (95% CI), months** | 10.6 (4.1, 12.0) | 13.6 (immature) ᵇ |

ᵃ Not evaluable in Cohort B: 2 patients without post-baseline imaging and 1 patient who received additional antitumor therapy of unknown start date. ᵇ Overall survival for Cohort B was immature at data cutoff (15/30 deaths); the 95% CI upper bound was not reached. CI, confidence interval; CR, complete response; DCR, disease control rate; DOR, duration of response; NE, not evaluable; NR, not reached; ORR, objective response rate; OS, overall survival; PD, progressive disease; PFS, progression-free survival; PR, partial response; SD, stable disease.

**Table S3. Grade ≥3 TRAEs attributed to finotonlimab (SCT-I10A)**

| **Event** | **Cohort A (N=11)** | **Cohort B (N=30)** | **Total (N=41)** |
| --- | --- | --- | --- |
| **Grade ≥3 TRAE** | 1 (9.1) | 14 (46.7) | 15 (36.6) |
| Hypomagnesemia | 0 | 4 (13.3) | 4 (9.8) |
| Hypocalcemia | 0 | 3 (10.0) | 3 (7.3) |
| Hypokalemia | 1 (9.1) | 2 (6.7) | 3 (7.3) |
| Type 1 diabetes mellitus | 0 | 1 (3.3) | 1 (2.4) |
| Diabetic ketoacidosis | 0 | 1 (3.3) | 1 (2.4) |
| Hyperkalemia | 0 | 1 (3.3) | 1 (2.4) |
| Acneiform dermatitis | 0 | 1 (3.3) | 1 (2.4) |
| Rash | 0 | 4 (13.3) | 4 (9.8) |
| Maculopapular rash | 0 | 1 (3.3) | 1 (2.4) |
| Alanine aminotransferase increased | 0 | 1 (3.3) | 1 (2.4) |
| Aspartate aminotransferase increased | 0 | 1 (3.3) | 1 (2.4) |
| Lymphocyte count decreased | 0 | 1 (3.3) | 1 (2.4) |
| Hypertension | 0 | 1 (3.3) | 1 (2.4) |
| Interstitial lung disease | 0 | 1 (3.3) | 1 (2.4) |
| Anemia | 1 (9.1) | 0 | 1 (2.4) |

**Table S4. Grade ≥3 TRAEs attributed to SCT200**

| **Event** | **Cohort A (N=11)** | **Cohort B (N=30)** | **Total (N=41)** |
| --- | --- | --- | --- |
| **Grade ≥3 TRAE** | 3 (27.3) | 21 (70.0) | 24 (58.5) |
| Hypomagnesemia | 2 (18.2) | 14 (46.7) | 16 (39.0) |
| Hypocalcemia | 0 | 2 (6.7) | 2 (4.9) |
| Hypokalemia | 1 (9.1) | 1 (3.3) | 2 (4.9) |
| Acneiform dermatitis | 0 | 2 (6.7) | 2 (4.9) |
| Rash | 0 | 4 (13.3) | 4 (9.8) |
| Maculopapular rash | 0 | 1 (3.3) | 1 (2.4) |
| Alanine aminotransferase increased | 0 | 1 (3.3) | 1 (2.4) |
| Aspartate aminotransferase increased | 0 | 1 (3.3) | 1 (2.4) |
| Lymphocyte count decreased | 0 | 1 (3.3) | 1 (2.4) |
| Hypertension | 0 | 1 (3.3) | 1 (2.4) |
| Paronychia | 0 | 1 (3.3) | 1 (2.4) |
| Anemia | 1 (9.1) | 0 | 1 (2.4) |

Footnote (Tables S3 and S4): A single adverse event could be attributed by the investigator to more than one study drug; therefore Tables S3 and S4 are not mutually exclusive, and their totals do not sum to the overall number of grade ≥3 TRAEs (25 of 41). TRAE, treatment-related adverse event.

**Table S5. TRAEs occurring in ≥5% of patients (finotonlimab plus SCT200)**

| **Adverse event** | **Any grade, n (%)** | **Grade 3 or 4, n (%)** |
| --- | --- | --- |
| **Any TRAE** | **40 (97.6)** | **25 (61.0)** |
| Hypomagnesemia | 26 (63.4) | 16 (39.0) |
| Rash | 17 (41.5) | 4 (9.8) |
| Acneiform dermatitis | 15 (36.6) | 2 (4.9) |
| Hypothyroidism | 12 (29.3) | 0 |
| Aspartate aminotransferase increased | 9 (22.0) | 1 (2.4) |
| Paronychia | 9 (22.0) | 1 (2.4) |
| Anemia | 9 (22.0) | 1 (2.4) |
| Alanine aminotransferase increased | 8 (19.5) | 1 (2.4) |
| Infusion reaction | 8 (19.5) | 0 |
| Hypoalbuminemia | 6 (14.6) | 0 |
| Hypocalcemia | 6 (14.6) | 3 (7.3) |
| Oral mucositis | 6 (14.6) | 0 |
| Maculopapular rash | 5 (12.2) | 1 (2.4) |
| Conjunctivitis | 5 (12.2) | 0 |
| Proteinuria | 5 (12.2) | 0 |
| Hematuria | 5 (12.2) | 0 |
| Hypokalemia | 4 (9.8) | 3 (7.3) |
| Thyroid stimulating hormone decreased | 4 (9.8) | 0 |
| Pruritus | 3 (7.3) | 0 |
| Exfoliative dermatitis | 3 (7.3) | 0 |
| Hyponatremia | 3 (7.3) | 0 |
| Lactate dehydrogenase increased | 3 (7.3) | 0 |
| Platelet count increased | 3 (7.3) | 0 |
| Creatine phosphokinase increased | 3 (7.3) | 0 |
| Oral ulcer | 3 (7.3) | 0 |
| Asthenia | 3 (7.3) | 0 |
| Type 1 diabetes mellitus | 1 (2.4) | 1 (2.4) |
| Diabetic ketoacidosis | 1 (2.4) | 1 (2.4) |
| Hyperkalemia | 1 (2.4) | 1 (2.4) |
| Lymphocyte count decreased | 1 (2.4) | 1 (2.4) |
| Interstitial lung disease | 1 (2.4) | 1 (2.4) |
| Hypertension | 1 (2.4) | 1 (2.4) |

n, number; TRAE, treatment-related adverse event. Percentages are based on the safety population (N = 41). Any-grade events occurring in ≥5% of patients are listed; lower-frequency grade 3/4 events are also shown.

**Table S6. Post-progression anti-cancer treatments after finotonlimab plus SCT200**

| **Treatment category** | **Cohort A (N = 11)** | **Cohort B (N = 30)** |
| --- | --- | --- |
| **Patients receiving subsequent anti-tumor therapy** | 7 (63.6) | 11 (36.7) |
| Medication therapy | 7 (63.6) | 9 (30.0) |
| Anti-EGFR monoclonal antibody | 3 (27.3) | 1 (3.3) |
| Anti-PD-1/PD-L1 monoclonal antibody | 3 (27.3) | 4 (13.3) |
| Chemotherapy | 4 (36.4) | 7 (23.3) |
| Tyrosine-kinase inhibitor | 2 (18.2) | 1 (3.3) |
| Traditional Chinese medicine | 0 | 2 (6.7) |
| Clinical trials | 2 (18.2) | 0 |
| Non-drug therapy | 0 | 2 (6.7) |
| Surgery | 0 | 1 (3.3) |
| Radiotherapy | 0 | 1 (3.3) |
| Unknown | 0 | 1 (3.3) |

Categories are not mutually exclusive.
